# Experimentation or projectification of urban change? A critical appraisal and three steps forward

**DOI:** 10.1186/s42854-021-00025-1

**Published:** 2021-09-29

**Authors:** Jonas Torrens, Timo von Wirth

**Affiliations:** 1 Faculty of Industrial Engineering and Innovation Sciences, Eindhoven University of Technology, Eindhoven, The Netherlands; 2 Erasmus University Rotterdam, Erasmus School of Social and Behavioural Sciences, Dutch Research Institute for Transitions (DRIFT), Rotterdam, The Netherlands

**Keywords:** Urban experimentation, Projectification, Experimental governance, Urban transformation, Sustainability transitions

## Abstract

Urban experimentation has proliferated in recent years as a response to sustainability challenges and renewed pressures on urban governance. In many European cities, diverse and rapidly changing experimental forms (e.g. urban living laboratories, pilots, trials, experimental districts) are becoming commonplace, addressing ambitious goals for smartness, circularity, and liveability. Academically, there is a growing concern for moving beyond the focus on individual experiments and the insistence on upscaling their primary transformation mechanism. However, the phenomena of ‘projectification’ – whereby project-based forms of organising have become ubiquitous, shaping expectations about experimentation – is increasingly perceived as a barrier. Nevertheless, how specifically experimentation and projectification intersect remains unclear. Our theoretical perspective examines how the widespread tendency towards projectification shapes urban experimentation and the potential implications for urban transformations. It problematises the current wave of experimentation and how it contributes to the *projectification of urban change processes*. We present three steps to redress this issue and indicate directions for future research.

## Policy and practice recommendations

•Urban experimentation is happening in contexts where the project-logic is taken for granted, with added pressure from funding requirements for project-organisation
•‘Projectification of urban change processes’ is a self-reinforcing mechanism that induces short-termism and unambitious incrementalism
•Practitioners and scholars need to discern experiments and projects, invest in learning from existing projects, and develop hybrid infrastructures for learning

## Introduction

Urban responses to societal challenges are increasingly experimental (Bulkeley and Castán Broto, 2013; Evans et al., 2016; Fuenfschilling et al., 2019; Torrens et al., 2018; von Wirth et al., 2019). The nature and arrangements of multi-level governance surrounding climate change and sustainability have tended to disperse authority and multiply the capacities involved – enrolling many more actors and initiatives and displacing the assumption of centralised control underpinning modernist planning and state practices. In this context, urban experimentation has emerged as a means through which such actors attempt to ‘navigate and make sense of the present whilst also giving concrete form to particular visions of the future’ (see Bulkeley et al., 2015, 2019). At the same time, local governments and civil society actors around the world are grappling with a barrage of challenges – including the climate emergency, the aftermath of the COVID-19 pandemic, and the persistence of socio-economic exclusion and inequalities (McPhearson et al. 2021; Acuto et al., 2018; Alberti, 2017). These ‘wicked problems’ defy straightforward definitions and complete solutions and demand an exploratory stance and iterative approach to imagining, intervening and collective sense-making (Harris et al., 2010; Rittel and Webber, 1973). That context has inspired discussions about a novel mode of ‘governing through experimentation’ that could create unique opportunities and political implications for urban change (Bulkeley and Castán Broto, 2013; Raven et al., 2017).

Over the last decade, a ‘first wave’ of urban experimentation oriented towards sustainability manifested in various forms. These include climate change experiments (Castán Broto and Bulkeley, 2013; Hoffmann, 2011), demonstration-oriented pilots (Heiskanen et al., 2017; Ryghaug et al., 2019), urban living labs (Bulkeley et al., 2016; Marvin et al., 2018; Voytenko et al., 2016; von Wirth et al., 2020), experimental districts (Fitzgerald and Lenhart, 2016), and grassroots initiatives engaging with tactical urbanism and other temporary activities (Gernert et al., 2018; Håkansson, 2018). Urban studies and transition scholars have strived to conceptualise the foundations of this phenomenon (Evans, 2016; Evans and Karvonen, 2014; Karvonen and van Heur, 2014) and understand the implications of distinctive designs and configurations of experiments (e.g. Bulkeley et al., 2018; Raven et al., 2017). This debate has exchanged much with the sustainability transitions research on socio-technical experimentation (Berkhout et al., 2010; Sengers et al., 2019).

Despite enthusiasm around urban experimentation, the rapid proliferation and diversification of experiments force practitioners and academics to grapple with experimentation’s multiplicity, with both positive and negative implications. Processes of (urban) experimentation are, *by design*, temporary, situated, and organised with specific learning or innovation objectives in mind, and operate amidst uncertainty and ambiguity that challenge strict planning and straightforward implementation (Evans et al., 2016; Sengers et al., 2019a; Turnheim et al., 2018). Whereas initial studies focused on ‘scaling’ isolated experiments, Evans et al. (2016) observed a shift: scholars and practitioners are ‘moving past isolated experiments to consider how more long-term and varied modes of experimentation can stimulate broader urban transformation’ (p.10). This brings a need for ‘understanding the durability and multiplicity of experiments within their broader urban context’ (p.10). Multiplicity is, in these terms, potentially accelerating urban transformations.

Notwithstanding, recent debates among practitioners, researchers, and funders have highlighted the issue of ‘projectification’ as a potentially harmful characteristic and consequence of the first wave of urban experimentation. For instance, the discussion held at the 2019 Urban Transitions Pathways Symposium, organised by JPI Urban Europe,^1^ highlighted three related issues that could hinder the transformative potential of the current wave of experiments: projectification, fragmentation of governance capacities and discontinuity.

However, it remains unclear what counts as projectification, how it relates to experimentation, and what are its consequences. For instance, during that event, the term indicated that many experiments were initiated and run as projects, hindering learning, aggregation or scaling, duplicating activities and limiting thed scope for ‘systemic’ or ‘structural’ impact. Meanwhile, the literature on ‘projectification of funding’ refers to constraints in the duration and requirements of funding for sustainability initiatives inducing short-termism (Borgström et al., 2016; Ehnert et al., 2018). But there are also parallels with ‘projectified politics’ (Sjöblom et al., 2013) or ‘projectified governance’ (Munck af Rosenschöld, J., 2019; Munck af Rosenschöld and Wolf, 2017), where the prevalence of temporary forms of organising in the public sector reshapes how governments engage in societal issues (Hodgson et al., 2019). Hence, there is a need to examine the relationship between urban experimentation and projectification, which remains ambiguous and cluttered with definitional issues.

In this theoretical perspective, we address this ambiguity by examining the relationship between urban experimentation and different facets of the phenomena of projectification. We ask *how the widespread tendency towards projectification shapes urban experimentation and what are the potential implications for urban transformations.*

To begin, we briefly introduce two perspectives on urban experimentation (cf. Bulkeley 2019), which influence how multiplicity is understood. We then discuss how the experimental and project logic differ (Tables 1 and 2). Next, we argue experimentation is taking place in contexts where a project logic is prevalent through different forms of projectification, contributing to the process we label as the projectification of urban change processes. Finally, in section three, we indicate three initial steps for redressing these issues and conclude with promising avenues for future research.

**Table 1:** Distinctiveness of urban experimentation

**Urban experimentation** relates closely to the notion of ‘reflection in action’, whereby ‘to experiment is to act in order to see what action leads to’ (Schön, 1983, p. 144). Compared to other forms of urban development or policy, urban experimentation comprises a variety of experimental logics that share an inclination for learning from real-world interventions (Evans et al., 2016), with activities situated in real-world places; oriented towards producing changes with an emphasis on improvement or transformation; and an embrace of contingency and uncertainty (Karvonen and van Heur, 2014). Each of these ‘accomplishments’ can be organised in diverse ways (Bulkeley et al., 2019), leading to seemingly contradictory ideas of what constitutes experiments. Crucially, a processual perspective on experimentation is emerging in the literature that moves beyond ‘the context of individual experiments or collections of experiments to conceive of urban experimentation as a process that materially embeds priorities, that seeks to make them durable, through experimentation in particular places’ (Hodson et al., 2018). The current wave of experimentation has a distinctively urban character and is linked to efforts to deal with the complexities, uncertainties and contestations of ongoing urban transformation processes
Reviews mapping the rationales informing experimentalism highlight how diverse experiments can be. For instance, Sengers et al. (2019) identified five conceptualisations within the transitions’ perspective, differing in normative orientation, theoretical foundation, analytical emphasis, and main actors involved. Caniglia et al. (2017), writing from a sustainability science perspective, proposed a six-fold typology, differentiating the focus being either on problems or solutions, and three levels of control: full control, participatory control, and no control. However, that perspective foregrounds the production of evidence for decision-making, contrasting with the idea that experimentation aims to induce changes and improvements in the real world. Ansell and Bartenberger (2016), in turn, highlight distinct logics that can guide experimentation. Those authors distinguish *controlled experimentation*, based on highly controlled interventions aimed to test particular hypotheses in a deductive manner; *Darwinian experimentation*, oriented towards generating variety from which best practices can be selected through an inductive approach; and *generative experimentation*, which applies an abductive logic to interactively redesign and refine a prototype until it meets stakeholders expectations. Crucially, they highlight that learning generated through experiments is not only scientific or technical, but also ‘political learning’, by which ‘stakeholders may alter their preferences, goals, frames, and commitments’ (Ansell and Bartenberger, 2016)
Each of these typologies hints at a very rationalist and neutral process of designing experiments. In practice, however, how exactly experiments are set up depends on a contested process of negotiating priorities, epistemological assumptions, and normative goals while trying to create viable setups. As a result, experiments end up enrolled into varying processes of change. For instance, they could be used to generate ‘single loop’ learning about improving performances or particular artefacts or services that conform to existing rules (e.g. testing a smart energy meter); ‘double loop’ learning that inquires the rules and structures of a given system and prompts reforms (e.g. exploring options for energy retrofit with provision models such as community energy); or ‘triple loop’ learning, seeking to transcend and transform such rules and open up previously unimagined possibilities with new values and principles (e.g. establishing self-governed post-capitalist eco-settlements) (Waddel, 2016, cited in Fazey et al., 2018). However, only some of these various forms are formally recognised and labelled experiments or labs, which privileged certain forms of action and certain forms of knowledge as desirable and legitimate, constituting a political process with biases and normative assumptions (Savini and Bertolini, 2019)

**Table 2:** Projectification and challenges of projectified contexts in urban change

With **projectification**, we refer to the multidimensional phenomenon by which the project logic becomes the prevalent way of organising activities in diverse life domains and urban change processes in particular Projects can be defined in ideal and instrumental terms by a combination of a focus on plannable and unique tasks, involving complex or interdependent activities, subject to evaluation on a predetermined time frame, and with pre-specified performance criteria (Packendorff, 1995). Their appeal lies in being perceived as a ‘controllable way of avoiding all the classic problems of bureaucracy’ faced by routinised forms of organising (Packendorff and Lindgren, 2014; p.7)
Project management is often presented as a collection of tools, which promises clarity, order and control via standardised procedures (Packendorff and Lindgren, 2014). However, according to Brulin and Svensson (2011), strict adherence to such a ‘project logic’ is a short-sighted impediment for addressing sustainability challenges in the long term
Project-based forms of organising partially overlap with experiments' attributes, e.g. in their ability to provide temporary and contextually rich opportunities for learning. As Hodgson et al. (2019) argued, project-based arrangements are often considered as ‘attractive and relatively cheap ways to ‘test out’ or roll out new ways of working’ (p.4) and encouraging bottom-up innovation. Thus, they are seen as a desirable ‘vehicle for policy change’, with’a temporal desynchronisation between ongoing public sector activities and the intensive, transformation work of the policy project’, thus creating a ‘state of exception’ (p.134)
Project-based forms are also prevalent in urban change, for instance, in municipalities’ and utilities’ operations, especially in their attempts to induce institutional change (Munck af Rosenschöld, 2019). In the urban context, various project types are implicated (e.g. urban renewal and regeneration projects, infrastructure megaprojects) in a neoliberal political turn that shifts who decides the city's future
As Swyngedouw et al. (2002) critically observed, ‘planning through ‘urban projects’ has indeed emerged as the main strategy to stimulate economic growth and to ‘organise innovation’, both organizationally and economically (p.562). However, this is not simply an organisational but a political issue, because the ‘the imagin(eer)ing of the city’s future [is being] directly articulated with the visions of those who are pivotal to the formulation, planning, and implementation of the project’ *(Id., p.563).* Thus, projectification is politically fraught in that it cannot be disentangled from the interests and political positions of the involved project actors while also overlooking or excluding other interests and positions

## Unpacking urban experimentation and projectification

What is meant by urban experimentation remains a controversial issue. The literature on (urban) experimentation comprises various approaches and methods (Bulkeley and Castán Broto, 2013; Caniglia et al., 2017; Evans et al., 2016; Torrens et al., 2019). That is expected because experimentation is mobilised in diverse settings and underpinned by various epistemic traditions. A similar issue concerns projectification; it has been reported in different sectors and domains, with distinct consequences. Scholars attribute these phenomena to a range of causes; some suggest they are intrinsically connected. Nevertheless, the distinctions and similarities between experiments and projects are often muddled. If left unaddressed, this analytical conflation undermines the transformative potential of experiments.

Rather than simplifying this situation with a narrow and tight definition of urban experiments and projects, we favour a more discerning understanding of the wider spectrum of urban experimentation and projects may come to represent (see Tables 1 and 2).

### Urban experimentation: managed scaling or generative multiplicity

Considering the broader process of urban experimentation mentioned above, two perspectives deserve further attention according to Bulkeley (2019), one of the field’s pioneers. A) Conceiving experimentation as a means to translate and test policy goals (in pilots or labs) to generate learning and increase the capacity of networks of actors aiming to take those interventions’at scale’ (e.g. citywide) and enable transitions. B) Regarding ‘experimentation as a new – messier and provisional—mode through which governing is taking place, potentially replacing urban planning and policy as a means through which urban sustainability is realised’ (p.23). Arguably, these two perspectives diverge on whether experimentation centres on managing scaling and whether their proliferation is problematic.

Building on the first perspective, which sees experimentation as ‘a process of developing and trialling innovations as well as the institutions that nurture and scale them over time’ (Fuenfschilling et al., 2019, p. 220), numerous studies categorised the processes which mediate local experiments' impact beyond their initial context of application (e.g. Brown and Vergragt, 2008; Naber et al., 2017; Nevens et al., 2013; von Wirth et al., 2019). This includes attention to the impacts of urban living labs (von Wirth et al., 2019), and efforts to understand the barriers to scaling (Dijk et al., 2018) and the institutional arrangements enabling experimentation (Raven et al., 2019). A strand of the literature centres on the specific outputs and outcomes of experiments and the ‘embedding mechanisms’ that leads to persistent impacts ‘beyond’ the individual experiment (see Turnheim et al., 2018, Sengers et al., 2021). In a recent review, Lam et al. (2020) identified eight such ‘amplification processes’, highlighting the possibilities of a) prolonging or accelerating the impact of individual initiatives, b) extending the impact to more people or places, c) changing institutional structures, values and mindsets (amplification within, out, and beyond, respectively). These studies have expanded the analytical vocabulary for analysing the transformative potential of experiments.

Even from a ‘classical’ transition theory perspective, it is problematic to overemphasise upscaling or focus on individual experiments. The transitions literature is premised on the evolutionary nature of experimentation, with multiple activities happening in parallel, recombining and evolving, allowing for the exploration of complex problems’ and solutions’ framings. Numerous studies have centred on learning and aggregation of the knowledge generated across multiple experiments to create more robust practices and a gradual build-up of generic responses, best practices and standards (Geels and Deuten, 2006; Geels and Raven, 2006; Raven, 2007; van den Bosch and Rotmans, 2008; van Mierlo, 2012). They also stress the importance of articulating expectations surrounding experiments for defining or letting emerge particular guiding visions and the role of intermediaries as agents contributing to replicating experiments in different locations and circulating the knowledge (Barnes et al., 2017; Moss, 2009; van Lente et al., 2003). In this sense, transition studies highlight that multiplicity in experimentation can be generative when occurring in conjunction with other processes and situations (e.g. favourable macro-trends). Without those processes, it is improbable that experiments will—in and of themselves—scale to produce substantial shifts towards more sustainable trajectories. In this vein, Fuenfschilling et al. (2019) call for more attention to how experimentation can affect institutional change, become institutionalised or deinstitutionalise entrenched socio-technical configurations (p.220).

More pointedly, Bulkeley (2019) cautions, a narrow focus on scaling (or amplification for that matter) is misplaced: at best, it ‘gives a partial reading of the potential of experimentation, and at worst puts us on the wrong track altogether’ (p.31). Of course, there is probably scope to better design and conduct experimentation to increase their chances for success and impact. But to focus narrowly on those questions neglects the already messy and contingent character of urban governance.

As an alternative to the scaling-centric perspective, Hodson et al. (2017) highlight the potential for a more generative form of multiplicity, underpinned by the co-occurrence, competition, and complementarities along three dimensions. First, the presence and formation of ‘new configurations of multiple socio-technical experiments and existing systems’. Second, interactions with ‘multiple forms of urban governance’ conditioning and shaping experimental processes. Third, the effect of ‘multiple understandings of urban sustainability’ mediating these experimental processes. Foregrounding multiplicity requires a shift from focusing on scaling or amplifying tightly-bound individual experiments to the ongoing processes of reconfiguration that are triggered through experimental processes ‘assembling technologies, social interests, and new modes of governance into place-based configurations and learning about these processes of embedding an infrastructure or a scheme in a particular place’ (id. p.6).

Multiplicity also seems unavoidable; even attempts to increase the coherence of experimentation by formalising a method or approach—as with urban living labs—generate diverse configurations adapted to institutionally and politically specific settings (Bulkeley et al., 2019; Raven et al., 2017). Even when local governments adopt a proactive stance (Mukhtar-Landgren et al., 2019), they can pursue multiple pathways in parallel. Effectively, some expressions of experimentation may be valuable in part because they hold these dilemmas open for negotiation and contestation. Urban Living Labs, for example, ‘sit at the intersection between the more ‘temporary’ – with its multiple and shifting actors – on the one hand, and the ‘permanent’ organisation’ – on the other (Kronsell and Mukhtar-Landgren, 2018).

When multiplicity is considered in this way, a new picture emerges in which experiments become ‘critical means through which governing as normal takes place’ (Bulkeley and Castán Broto, 2013, p. 363). Bulkeley (2019) points towards experiments as integral to a messier form of governance, not aimed at controlling and scaling the outcomes of experiments. Instead:

‘*Experimentation serves to open up existing configurations, subjecting the logics, techniques, values, visions, practices and routines of infrastructural provision, consumption, risk calculations and so on to contestation and reworking. In this sense, experimentation is necessarily contradictory, requiring the bringing into relation of different socio-material orders and their navigation* (Bulkeley, 2019)

In this perspective, ‘experiments become embedded (…) not through scaling and transfer but through the gradual replacement of existing modes of governance’ (Karvonen, 2018), in a prospective ‘city of permanent experiments’ marked by an embrace of uncertainty (through reflexivity and responsiveness), the development of *recurring* learning aimed at the enactment of desired futures, and the potential fragmentation (or ‘spatial delineation’) of the city into distinct experimental districts (id.). Hence, this strand portrays the multiplicity of experimentation not as a barrier to attempts to generate wider impact, but as a potentially generative feature, with the city construed as’a provisional achievement (…) that is always ‘in the making’” (id., p.206).Given the previous arguments, we define the *generative multiplicity* of experimentation as pathway for the evolution of urban experimentation in a given context, premised on sustaining and culturing plural variations of experiments simultaneously, bringing about new forms of contestation and contradiction to stimulate higher-order learning processes and transformation of socio-material configurations. We consider multiplicity in urban experimentation as inherently and overtly political, as multiple experiments potentially challenge existing distributions of power and agency while also being prone to the risks of capture and of reinforcing current power asymmetries and inequalities.

Despite these insights from the urban experimentation literature, the recent experience with the current wave of experimentation in cities falls short in each of these aspects, prompting concern over methodologies employed and ensuing ‘projectification’. For instance, when observing the governance of living labs, Leminen et al. (2012) reported a tendency to short-term focus on organisational needs and tensions with applying project management tools that suggest linear, sequential thinking for a context, which instead asks for reflexivity and systems orientation. Others have observed that experimental ‘failures’ (as in not meeting the expected outcomes of participants) are in themselves hindrances to long-term learning (Collins, 2020). Furthermore, the prevalence of an experimental logic is possibly reshaping urban governance significantly. The multiplicity described above is related to a highly idiosyncratic approach to learning, articulating expectations and intermediation. Few experiments draw on comprehensive transition frameworks.

In sum, the literature on urban experimentation shows tension between two distinct conceptions of the transformative potential of experiments, either emphasising the prospect of managing the scaling up and amplifying experiments’ outcomes or rekindling urban governance itself. In the former, the proliferation (beyond specific efforts to replicate initiatives) of experiments is seen as a sign of undesirable fragmentation and dilution of efforts, diverging from stated goals and visions, which needs to be redressed to increase the chances of systemic impact (e.g. widespread adoption and institutionalisation). In the latter, experimentation is perceived as a potentially generative and transformative new means of governance precisely because of its multiplicity, not despite it. It creates affordances to reconfigure, question and renegotiate urban change in more provisional, contested and unruly ways. These perspectives diverge in how they regard projectification.

### Unpacking projectification

In debates about the proliferation of urban experiments, ‘projectification’ has been used to raise concerns about the problems caused by relying on temporary, situated interventions to address persistent, systemic challenges. This term draws attention to the ways experiments are being shaped by a longstanding project logic that pervades both urban change and sustainability. However, projectification is not a new phenomenon (see Table 1).

Projects addressing sustainability have also become ubiquitous. Brulin and Svensson (2011) outline the contours of the project logic, highlighting why it hinders long-term sustainability.

*‘Projects are based on rational thinking, where goals are set up, funds chosen, activities implemented, and results attained. The starting point is consensus thinking where opposition and conflicts are conspicuous by their absence. There is an underlying thought that the best solution and goals are unambiguous, not full of conflict or contradictions. Surroundings are regarded as stable and the future as predictable. Results are assumed to be capable of being transferred irrespective of situation, and processes are not regarded as important for results.’ (*Brulin and Svensson, 2011*, p.11)*

Although seemingly desirable for those seeking controllable scaling, these characteristics contrast dramatically with the generative perspective and its provisional, unruly and contested depiction of urban change.

To state that experimentation causes projectification is to miss the point. Instead, it is crucial to consider the issues that arise when conducting urban experimentation in governance settings and urban contexts *already highly projectified*. Projectification, seen this way, co-constitutes the practices of urban experimentation. We argue that nesting urban experiments in a governance setting dominated by the project logic biases the forms of experiments (and impacts) that emerge and receive support. This bias narrows the scope for experimentation and influences which urban development pathways are pursued (e.g. foregrounding scaling of marketable innovations). That may reinforce a lock-in of current urban change processes, diluting more radical transformative efforts and learning opportunities.

These concerns are not exclusive to urban experimentation. The debate around projectification (of the public sector) provide insights into its implications. That body of literature discusses two dimensions of projectification (Hodgson et al., 2019; Packendorff and Lindgren, 2014). In a ‘narrow’ or ‘organisational sense’, projectification refers to restructuring organisations around projects as crucial operational units. In a ‘broad’ sense, it concerns the ‘more fundamental discursive spread of projects and related phenomena as they become embedded, naturalised and institutionalised across organisations, societies, and in everyday lived experiences’ (Hodgson et al., 2019, p. 3). Furthermore, as projects permeate not only organisations but private lives, the ‘projectification of everything’ may effectively represent a radical departure from a society fundamentally organised around hierarchy, activity, space, time and relations (Jensen et al., 2016).

In particular, Munck af Rosenschöld (2017, 2019) argues that we witness the emergence of different arrangements of ‘projectified governance’. These range from a) mechanistically applying projects with a focus on the exploitation of existing knowledge to address simple problems while still relying on permanent organisations, b) organically decentralising management of projects for supporting exploration, c) and adaptively combining exploration and exploitation in settings with interfaces between projects and networks. Hence, it is projectification and experimentation intersect in complex, context-specific ways. However, in attempting a first conceptual overview of how experimentation is co-constituted by the tendency towards projectification, three issues stand out.

First, infusing experiments with project logic may distort their aims and conduct, emphasising delivery and implementation, strict monitoring of quantifiable outputs, and the expectation of efficient operations in a controllable and cost-efficient manner (Brulin and Svensson, 2011; Munck af Rosenschöld and Wolf, 2017). These pressures further narrow the possibilities of political learning and hinder reflexivity and deliberation.

*Questions of what ‘should’ be done, which might ordinarily be subject to lengthy deliberation, become relegated behind the question of what can be delivered quickly, what can be rendered measurable in this timescale, and practical questions of how it might be implemented.* (Hodgson et al., 2019, p. 143)

In such contexts, running experimental processes as projects risks rendering the political as technical, depoliticising issues. By focusing on translating political goals into achievable milestones and treating these milestones as non-negotiables once projects start, this approach may shut down the need for contradiction, contestation and reworking highlighted by Bulkeley (2019). Under a strict project logic, related issues (e.g. resistance of stakeholders) are treated as barriers to completion to be managed as risks. It may also privilege initiatives that may ‘fit-and-conform’ into existing structures, as the business of ‘stretching-and-transform’ those structures is often unruly and untimely (Smith and Raven, 2012). Hence, ‘*unambitious incrementalism’* may become prevalent, which privileges the achievable over the necessary.

Second, the widespread adoption of projects-forms in a ‘state of exception’ can exacerbate the sense of *precarity and ephemerality* of sustainability or transformational efforts without inducing changes to public sector organisations' routine operations or attaining long-term societal goals. However, the delegation of authority implied in projectification means previously political decisions become more and more decentralised but also less accountable. Concerning urban experimentation, this raises the prospect of ‘organised irresponsibility’ (Beck, 1998); a situation in which (urban) actors experiment and learn in an open-ended manner, while there is no one held accountable or responsible for the outcomes and continuity of the efforts (von Wirth et al., 2019). This results in a lack of directionality unless other forms of collective leadership emerge.

Third, the *‘projectification of funding’* (c.f. Borgström et al., 2016) induces *short-termism* and may hinder epistemic and political learning. Research and innovation funding, which sustain many experiments, demand projects as the organising form (e.g., provided through the Horizon Europe, JPI Urban Europe, Interreg program). Experimental initiatives largely depend on external funds with limited duration and no guarantee of renewal, provided through fragmentary and unstable multi-level governance arrangements (Ehnert et al., 2018). Innovation funding often requires that initiatives demonstrate self-sufficiency early on, *limiting the participation* of grassroots initiatives based on voluntary and not-for-profit organisations and privileging start-ups with ‘bankable’ business models. In the public sector, the projectification of funding means little support is available for intermediary or boundary-spanning functions that may embed experimentation. It keeps experiments detached from routine organisational processes, with precarious arrangements unable to support continuity and diversity of experimental approaches. Policy officers are often scraping together funds to keep programmes running and are often *unable to ensure the follow-up* of even the most promising initiatives (e.g. Torrens et al., 2018). As such, public authorities tend to anchor an urban experiment only loosely, with non-binding commitments (e.g. a single policy officer responsible for running experiments and reporting results). This situation generates unmet learning promises and disappointment, with considerable ‘institutional or organisation amnesia’ (Pollitt, 2000), the ‘intentional or unintentional ways in which government agents and organisations (…) no longer remember or record policy-relevant lessons from the past’ (Stark and Head, 2019, p. 1526). Projectification of funding may be present even when internal funding is available because of requirements to adhere to project-based accountancy standards.

In sum, the widespread projectification prevalent in society co-constitutes and shapes the trend towards urban experimentation through projectified governance and projectified funding. In such contexts, experiments start adhering to a project logic (becoming ‘projectified of experiments’) and further reinforce the other forms of projectification.

**Fig. 1:**
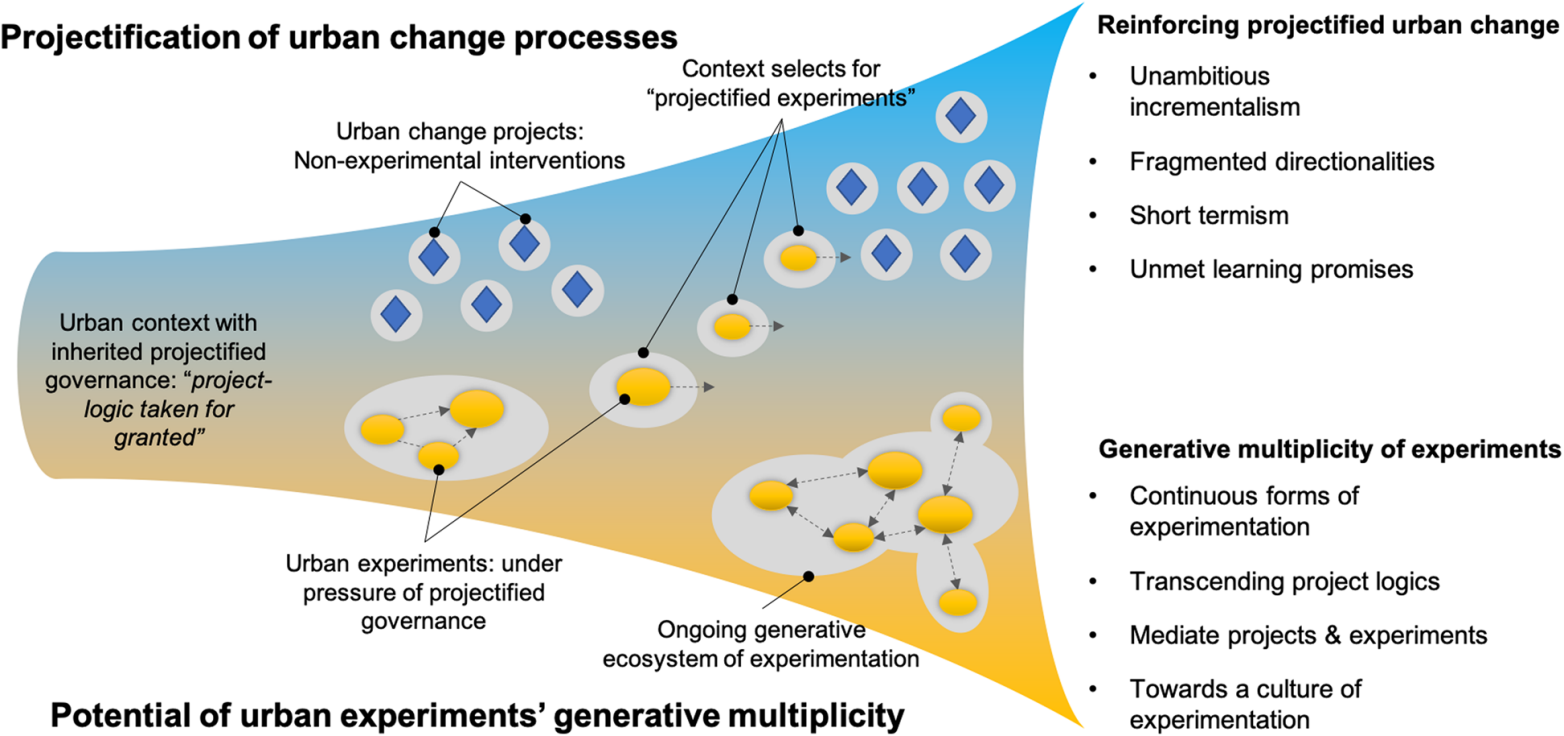
Schematic representation of how experimentation can evolve in projectified settings, either reinforcing the projectification of urban change or tending towards generative multiplicity

The resulting self-reinforcing dynamic gives rise to a pathway for the evolution of urban experimentation, marked by the *projectification of urban change processes* (Fig. 1). Tentatively, we define it as the *unreflexive reliance* on discrete, narrow-scope and time-bounded experimental interventions to deal with complex, multidimensional and persistent urban challenges, conforming to a narrow project logic, *without appropriate means* for learning, aggregation or reflexivity, which forecloses or hinders potential avenues for urban change.

An example is found in an earlier iteration of the experimental programme ‘Innovation for Physical Environment’ developed by the Utrecht province in the Netherlands (Sattlegger, 2020). There, the notion of sustainability transitions has initially informed the turn towards experimentation. Yet, the organisational culture and expectations were centred around projects. Experiments were then primarily thought of as innovation projects that could generate scalable solutions. Despite relative success in initiating multiple initiatives addressing the provinces’ challenges, the programme’s initial phase created various disconnected activities, which lacked follow-up and had limited institutional impact.

Contrastingly, settings in which experimentation is not pressured into conformity with the project logic can allow for urban change to tap into ‘generative multiplicity’, closer to what has been described by Hodson et al. (2017). In the Utrecht case, the province accumulated experience with varied forms of experimentation (Sattlegger, 2020) and sought to integrate experimentation to new demands for integrated visioning prompted by a new Environmental Act. The province is presently seeking to develop the means for a) embedding the capacity to experiment more closely to its core processes by creating a new community of practice, b) developing more sophisticated processes for learning from experiments and share those lessons internally, including bespoke forms of evaluation. That process is slowly taking place, forcing the organisation to explore and specify what experimentation as a mode of governance means for their challenges, building new competencies and processes along the way, eventually transcending the traditional project logic and its organisational routines.

The contrast between projectified settings and generative multiplicity is also evident when examining the long term development of urban experimentation in particular places. For instance, Torrens et al. (2018) traced the development of a favourable environment for experimentation with sustainability in Bristol (UK). Despite recurrent fears related to projectification, the proliferation of grassroots initiatives and strong intermediary organisations working alongside more traditional projects by the municipality gradually reconfigured the context to the point that it can sustain high degrees of experimentalism in multiple areas of sustainability (Barnes, 2015). However, Hodson et al. (2018), writing on the urban experimentation with mobility in Greater Manchester (UK), found evidence of significant learning from experiments, but with ‘inadequate institutional mechanisms for coordinating multiple experiments, capturing such learning and using such learning to systematically reshape conditions (for experimentation)’. In their case, those authors found ‘conditions shape experimentation’, but ‘experimentation only contributes weakly to shaping conditions’ (p.1495). Those findings are certainly context-dependant, but it is reasonable to expect that—in the absence of specific efforts to combat it—the projectification of urban change processes would affect most contexts.

## Redressing the projectification of urban change processes

To address the issues raised above, we identify three recommendations for the practice and research of urban experimentation.

- **Stop assuming, a priori, that experiments should work as projects.** Experiments may be run in multiple ways, many of which do not conform to projects' expectations. It is critical to determine, intentionally, what aspects of project management are ‘imported’ into handling experiments. Three issues demand attention. First, the emphasis on successful implementation and risk reduction introduces biases to experimentation. It moves away from more ambiguous or uncertain pathways, even when they promise sustainability or justice. It privileges particular forms of experimentation (e.g. policy pilots) to the detriment of others (e.g. grassroots experiments as part of transdisciplinary research settings). Second, evaluating and monitoring experiments against performance indicators is often problematic, focusing on easily measurable outputs and missing more qualitative outcomes of experimentation, such as reflexive learning, the emergence of new narratives, or the settling of deeply rooted disputes. Third, strategies used to provide coherence across projects may further reinforce the issues above. Deliberating about which aspects of project management are needed and which are to be avoided creates openings for more radical, open-ended forms of experimentation. But deliberation requires embracing uncertainty, conflict and contestation not as risks to be mitigated but as the *raison d’être* of experimentation. Hence, this step is in tension with organisational routines and professional norms of most city’s institutional environments—for instance, the planning bureau or maintenance department – that seek to minimise uncertainty and establish formal avenues for conflict resolution. Addressing this tension, implicitly and explicitly, when negotiating how experimentation is organised and establishing new internal norms is essential to ‘institutionalise’ experimentation without quenching its generative prospects. Some of these routines and norms (e.g. safety inspections and financial reporting) may remain non-negotiables that inform experiments’ design.
- **Strive to render traditional projects more experimental.** Experiments are not the only process through which organisations and communities learn. The multitude of projects that emerge in cities is a rich substrate to derive learning and insight. However, this substrate is often disregarded in a rush to establish novel initiatives. Even as experimentation proliferates, it remains a small part of cities' overall expenditure; for every experiment run, hundreds of projects go without dedicated efforts to learn and evaluate. They are often overrun by the start of new projects. Furthermore, where evaluation takes place, it is often summative, focused on checking goals' attainment, curtailing learning. Connecting formal experiments to the ongoing, routine activities and projects of cities is essential, a) to inform the agenda of what needs to be explored, studied, demonstrated; b) to identify synergies whereby experiments use the opportunities created by other projects (e.g. maintenance operations); and c) to capture unexpected discoveries uncovered by projects, which may go unreported otherwise.
- **Establish hybrid spaces that mediate between projects and experiments and permanent organisations.** Both projects and experiments usually operate with restrictive budgets and resources. They lack the resources to facilitate lengthy deliberation and learning processes or convene reflexive workshops and often privilege the dissemination of results. These efforts also require specific sets of skills and capacities that are not common among traditional project managers or more action-oriented individuals invested in ‘getting things done’. It would be ineffectual, expensive and unpractical to add ‘deep’ monitoring and reflexivity to every individual project and experiment without the appropriate support. Therefore, establishing reliable means to support such processes is paramount. Many questions or dilemmas conducive to deeper learning are not accessible at a project/experiment level and require cross-section examination of multiple instances (Potjer, 2020; van den Bosch and Rotmans, 2008). Establishing structures, processes or communities that facilitate cross-learning and translocal boundary-spanning enables much-needed reflections about place-specificity and replicability of lessons from several experiments. We emphasise hybridity for a) juxtaposing and recombining political and epistemic learning emerging from experiments and projects b) negotiating the tensions and dilemmas that arise in the conduct of experiments and projects, as well as aligning expectations for the involved stakeholders, c) supporting the forms of deliberation, visioning, and radical imagination otherwise suppressed by the operational pressures of individual initiatives (e.g. through ‘techniques of futuring’, see Hajer and Pelzer, 2018).

## Conclusions

This perspective examined the relationship between urban experimentation and different forms of projectification. We argued that the widespread reliance on project-based forms of organising influences and co-constitutes the current urban experimentation wave. However, pursuing experimentation in *projectified governance settings* is mired with challenges, which may contribute to depoliticising experiments and reinforce the ‘organised irresponsibility’ around long term sustainability transformations. In parallel, the *projectification of funding* limits what can be pursued experimentally. Meanwhile, conflating experiments and projects further entrenches the project logic, reinforcing the other forms of projectification. That combination creates the risk of projectification of urban change processes, which induces unambitious incrementalism, short-termism, lack of direction, lack of follow-up, and unmet learning promises. When this self-reinforcing dynamic is unmitigated, scepticism towards the transformative potential of experimentation is well justified.

However, we contend that the present debate on urban experimentation also outlines distinct avenues for harnessing experiments' generative multiplicity (Bulkeley, 2019; Hodson et al., 2017; Karvonen, 2018). These reframe concerns over the proliferation of experiments and highlight the potential for generative forms of ongoing, provisional and deeply political forms of urban experimentation that would significantly expand, if not transcend, the project logic's present confines and the myopic pursuit of scaling. For that, it is critical that we stop conflating projects and experiments, strive to render projects experimental, and find new ways to mediate between projects, experiments and permanent organisations.

For the urban transformations community, engaging with the criticism around projectification raises multiple political issues, such as the effects of neo-liberal agendas, privatisation of public spaces, and outsourcing of municipal expertise. But rather than just denouncing the projectification, our community should proactively develop new strategies to mitigate these issues while further exploring and harnessing the potentials of generative multiplicity in urban experimentation.

## Footnotes

1 JPI Urban Europe is the Joint Programming Initiative of the European Commission, a major funder of urban experiments in Europe, with a central role in the diffusion of the Urban Living Lab approach in response to sustainability challenges. JPI hosted the symposium in Maastricht (Netherlands) on 21–22 October 2019. It was attended by the main author and around 20 other experts in urban experimentation.
